# Prevalence and risk factors of vertebral compression fractures in female SLE patients

**DOI:** 10.1186/ar3104

**Published:** 2010-08-02

**Authors:** Katarina Almehed, Szabolcs Hetényi, Claes Ohlsson, Hans Carlsten, Helena Forsblad-d'Elia

**Affiliations:** 1 Department of Rheumatology and Inflammation Research, Sahlgrenska Academy at University of Gothenburg, Guldhedsgatan 10A, S-413 46 Göteborg, Sweden; 2 Department of Radiology, Sahlgrenska University Hospital, Sweden; 3 Current address: European Telemedicine Clinic, Torre Mapfre C/Marina 16-18, 08005 Barcelona, Spain; 4 Department of Internal Medicine, Sahlgrenska Academy at University of Gothenburg, Grönastråket 8, S-413 45 Göteborg, Sweden

## Abstract

**Introduction:**

Our objective was to determine the frequency of and factors associated with prevalent vertebral compression fractures in female systemic lupus erythematosus (SLE) patients attending rheumatologists in western Sweden.

**Methods:**

In this cross sectional study 150 women were included. They were examined with x-ray of thoracic and lumbar spine (Th4 to L4). A reduction of at least 20% of any vertebral height, assessed by Genant's semiquantitative method, was defined as a fracture. Bone mineral density (BMD) was measured by dual energy x-ray absorptiometry (DXA).

**Results:**

Median patient age was 47 years (20 to 82) and disease duration 11 years (1 to 41). Only 6 (4%) women had a history of clinical compressions whereas 43 (29%) had at least one radiological fracture each. The patients with at least one fracture at any site were characterized by older age (*P *< 0.001), being postmenopausal (*P *< 0.01), higher Systemic Lupus International Collaborative Clinics Damage Index (*P *< 0.05), lower BMD total hip and femoral neck (*P *< 0.05), more peripheral fractures (*P *< 0.01), medication with bisphosphonates (*P *<0.05) and calcium and vitamin D3 (*P *< 0.05). There were no significant differences regarding current or cumulative glucocorticosteroid dose between the groups. In logistic regression analyses high age remained as a risk factor of at least one vertebral fracture at any site whereas low BMD in total hip was associated with vertebral fracture in the lumbar spine.

**Conclusions:**

Radiological compression fractures are common but seldom diagnosed in SLE patients. High age and low BMD in total hip, but not in spine, was associated with vertebral fractures.

## Introduction

With improved treatment, long term morbidity like cardiovascular disease and fractures become increasingly important in managing systemic lupus erythematosus (SLE). Several studies have shown increased risk for peripheral as well as vertebral fractures in SLE patients compared to the general population [1-3]. Peripheral fractures are often easy to diagnose whereas vertebral compression fractures can be clinically silent [4] or be recognized as *ordinary *back pain by the patient and therefore overlooked [5]. Glucocorticosteroids, often used in the treatment of SLE, may not only increase the loss of bone [6] but also influence vertebral strength by affecting bone cell survival [7].

Low bone mineral density (BMD) is a known risk factor of fracture [8]. Since several other risk factors are hard to influence, much effort is given to find and pharmacologically treat patients with low BMD. SLE patients often have other concurrent risk factors of low BMD and fracture like glucocorticosteroid medication, early menopause [9,10] and sun avoidance with possible vitamin D deficiency [11,12]. Recent studies have shown that vertebral compression fractures are common in SLE patients in spite of normal BMD [2,13] indicating alternative mechanisms to the origin of compression fractures. It also points to the necessity of radiological examination in finding compressions of the spine.

The aim of this study was to establish the prevalence of clinically reported and radiological verified vertebral compression fractures in female SLE patients in western Sweden. We also wanted to look for risk elements associated with these fractures.

## Material and methods

### Patients

All patients with SLE attending the rheumatology clinics in Göteborg and Borås, Sweden, were identified from administrative registers and invited to participate in this cross-sectional study. The procedure of enrolment has previously been described in detail [14]. In short, 339 patients, 298 women and 41 men, were identified. There was a 70% reply frequency among the female patients. The main reason for women not to participate were not wanting or not being able to participate (*n *= 30) and not meeting the inclusion criteria of at least four of the 1982 American College of Rheumatology (ACR) classification criteria for SLE [15] (*n *= 18). One hundred sixty-three female patients were included in the study. Thirteen were excluded in this analysis because they lacked radiographs. Data regarding 150 patients were included and analysed. For each patient data on demographic and disease related variables like age, duration of disease, weight and height, medication, dietary calcium intake, smoking habits, physical activity and clinical fractures after the age of 25 were assessed by self administered questionnaires. Both low and high energy peripheral fractures were reported. Dietary calcium intake was calculated from information on average intake of cheese and milk. Exercise was recorded as "times per week with regular physical exercise" and it is therefore possible to get a median value of zero.

The Systemic Lupus Erythematosus Disease Activity Index 2 K (SLEDAI-2K) [16] was used to score disease activity. Disease damage was recorded according to Systemic Lupus International Collaborative Clinics/American College of Rheumatology Damage Index (DI) [17]. Cumulative corticosteroid intake was calculated by a thorough reading of all patients medical records. The same rheumatologist assessed all patients (KA). Glomerular filtration rate (GFR) was predicted using the Cockcroft and Gault equation [18]. GFR (ml/min) = (140-age) × weight (kg) × 1.04/S-creatinine(μmol/l).

### Assessment of vertebral compression fractures

Each patient underwent two lateral conventional radiographs, one of the thoracic and one of the lumbar spine. Due to a shift of radiology equipment during the study period, approximately half of the patients had analog and the other half had digital radiographs taken. The same radiologist (SH) evaluated all radiographs for prevalent vertebral fractures using Genant's semiquantitative method [19]. Thirteen vertebrae per patient (Th4 to L4) were assessable for all patients but one where 11 vertebrae were assessable. In short, the vertebrae were visually graded as normal (grade 0), mildly deformed (grade 1, approximately 20 to 29% reduction in anterior, middle and/or posterior height and a 10 to 20% reduction in an area), moderately deformed (grade 2, approximately 30 to 45% reduction of any vertebral height and a reduction in an area of 20 to 40%), and severely deformed (grade 3, >45% reduction in any vertebral height and >40% reduction in an area). For each patient the number of fractured vertebrae was counted. A vertebral severity sum was calculated by addition of the Genant grades (0 to 3) from 13 vertebrae for each patient.

A vertebra was considered fractured when it was mildly deformed, grade 1.

### Laboratory tests

Venous blood samples were taken after a one-night fast and analysed consecutively using standard laboratory techniques in the Department of Clinical Chemistry at Sahlgrenska University Hospital and at Borås Hospital.

### Bone mineral density (BMD) measurements

The lumbar spine (L2 to L4), non dominant hip (femoral neck and total hip) and non dominant distal forearm were measured by DXA, Lunar Prodigy densitometer, 12165 (GE Medical Systems GE Healthcare, Little Chalfont, Buckinghamshire, UK). The precisions for duplicate measurements were 0.9% for the lumbar spine, 0.5% for the left total hip and femoral neck and 2.8% for the radius.

### Ethical aspects

All patients gave informed written consent prior to participation and the study was approved by the Ethics Committee at Sahlgrenska Academy at University of Gothenburg, Sweden.

### Statistical analysis

Analyses were performed using SPSS version 12.0.1 (SPSS Inc., Chicago, IL, USA). Descriptive statistics are presented as median and range or mean and standard deviations (SD). All variables in Table 1, including serum ionized calcium, ESR and CRP, were tested with Kolmogorov-Smirnov's normality test. A T-test was used for comparison of normally distributed demographic and disease related variables and the Mann-Whitney U-test was used for not normally distributed variables between patients with and without vertebral fractures. A χ^2^-test was used to compare categorical variables. Significant variables were then entered in a logistic regression analyses as covariates and having one or several vertebral fractures scored 1 to 3 according to the Genant's method as dependent variable. A forward conditional method was used. A receiver operating characteristic (ROC) curve was then calculated with vertebral fracture as the state variable. All tests were two-tailed and *P *< 0.05 was considered statistically significant.

**Table 1:** Demographic and disease related variables in 150 female patients with SLE

Demographic variables	Value
Patient age (years)	47 (20 to 82)
Weight (kg)	67 (43 to 96)
Height (cm)	166 (145 to 182)
BMI (kg/m2)	24.5 (17.2 to 35.3)
Exercise/week	0 (0 to 7)
Smoking status	
Nonsmoker *n *(%)	59 (39)
Previous smoker *n *(%)	48 (32)
Current smoker *n *(%)	43 (29)
Years of smoking, current or previous	24 (0.5 to 48)
Menopausal status	
Premenopausal *n *(%)	67 (45)
Postmenopasal *n *(%)	81 (55)
Dietary calcium intake (mg/day)	467 (0 to 1,510)
**Disease variables**	
Disease duration (years)	11 (1 to 41)
SLEDAI-2K	5 (0 to 31)
DI	2 (0 to 11)
Haemoglobin (g/l)	131 (80 to 156)
ESR (mm/1 hr)	19 (2 to 125)
CRP (mg/l)	5 (3 to 79)
Creatinine (μmol/l)	88 (49 to 291)
Calcium, ionized (mmol/l)	1.22 (1.07 to 1.42)
Glucocortocosteroid (Prednisolone) user *n *(%)	78 (52)
Prednisolone dose (mg)	5 (2.5 to 35)
Glucocorticosteroid (Prednisolone) ever user *n *(%)	129 (86)
Cumulative Prednisolone dose (g)	11 (0.1 to 97.5)
Immunosuppressive drug user *n *(%)	80 (53)
Calcium and vitamin D *n *(%)	81 (54)
Bisphosphonates *n *(%)	21 (14)
Postmenopausal	
HRT *n *(%)	4 (5)
BMD lumbal spine (g/cm^2^) mean (SD)	1.12 (0.18)
BMD total hip (g/cm^2^) mean (SD)	0.93 (0.14)
BMD femur neck(g/cm^2^) mean (SD)	0.89 (0.14)
BMD radius total (g/cm^2^) mean (SD)	0.51 (0.08)
Patients with vertebral fracture *n *(%)	43 (29)
Vertebral fractures/patient	0 (0 to 11)
Sum of Genant's grading per patient	0 (0 to 22)
Patients with peripheral fracture *n *(%)	22 (14)

Values are medians and (range) when not indicated otherwise.

BMD, bone mineral density; BMI, body mass index; CRP, C-reactive protein; DI, Systemic Lupus International Collaborative Clinics/American Collage of Rheumatology Damage Index; ESR, erythrocyte sedimentation rat; HRT, hormone replacement therapy; SERM, selective estrogen receptor modulator; SLEDAI-2K, SLE disease activity index-2K.

## Results

### Demographic and disease related variables

The SLE patients attending this study did not differ significantly in age from those who were invited but did not participate. The general characteristics of the study population are shown in Table 1. Fewer than 10 patients were not Caucasian. The participants' ages ranged from 20 to 82 years. Sixty-seven (45%) were premenopausal. Eighty (53%) were on immunosuppressive drugs, 78 (52%) were treated with glucocorticosteroids and 50 (33%) were treated with both immunosuppressive drugs and glucocorticosteroids. One patient had end-stage renal disease while 17 (11%) had a calculated glomerular filtration rate (GFR) less than 40 ml/minute. Nineteen patients (13%) were treated with thyroid hormones because of low thyroid function. Thirty-one (21%), 13 (9%) and 9 (6%) of the women had osteoporosis according to the definition of WHO [20] in at least one, two and three or more sites, respectively. Osteoporosis in the radius total was diagnosed in 22 (15%) of the patients, in the lumbar spine 16 (11%), in the femoral neck 11 (7%) and in the total hip 9 (6%). Eleven patients had substantial vertebral osteoarthritis.

### Vertebral fractures

**Figure 1:**
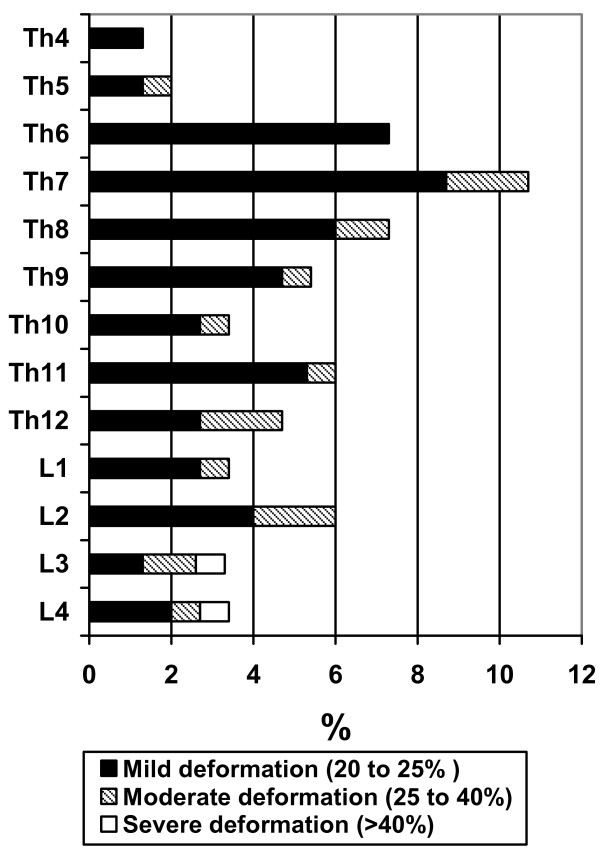
**Percentage of 150 female SLE patients with radiographic compression fractures per vertebral level**. Severity grade defined by Genant's method.

Forty-three women (29%) had at least one and 22 (15%) had at least two prevalent vertebral compressions each. Two patients had 10 and 11 compressions each. Thirteen (30%) patients with vertebral fracture had active treatment against osteoporosis, 11 were treated with bisphosphonates, 1 with hormone replacement therapy and 1 with selective estrogen receptor modulator therapy. Twenty-nine (67%) patients were medicated with calcium and vitamin D3. It was not significantly more common to have at least one vertebral fracture in a patient with current or former corticosteroid medication (36/129) as compared to corticosteroid naïve patients (7/21). Five out of six patients who reported knowledge of clinical vertebral fracture also had one or several radiological fractures. Thus, 38 (25%) patients had one or more asymptomatic or not diagnosed fracture. The proportion of patients with prevalent vertebral compressions increased with age but compressions were present in premenopausal ages as well, Table 2. Mild fractures, grade 1, were most common and represented 75 (79%) of all fractures. The fracture prevalence was highest in mid thoracic spine, Th6 to Th8, whereas compression severity was highest in lower lumbar spine, Figure 1.

**Table 2:** Prevalence of vertebral fractures in female SLE patients according to age

Age	Total patient number	Patients with any vertebral compression	Total number of vertebral compressions
Years	n	n (%)	n
20 to 29	17	1 (6)	1
30 to 39	23	4 (17)	4
40 to 49	41	11 (26)	17
50 to 59	42	14 (33)	37
60 to 69	15	6 (40)	13
70 to 79	11	6 (54)	21
80 to 89	1	1 (100)	2
	150	43	95

### Peripheral fractures

Twenty-two patients (15%) reported a total of 29 clinical peripheral fractures. There were 13 wrist, 8 ankle, 6 upper arm and 2 hip fractures reported. Seventeen (40%) of the patients with at least one vertebral compression fracture had experienced one or several peripheral fractures whereas 12 patients (55%) who had experienced a peripheral fracture also had at least one compression fracture.

### Risk factors of vertebral fractures

Patients with one or more vertebral compressions, grade 1 to 3, were compared with patients without any compressions regarding all demographic and disease related variables shown in Table 1. Variables significantly associated with compression fractures are displayed in Table 3. Current or cumulative doses of glucocorticosteroid medication were not significantly associated with vertebral fracture, neither was GFR nor thyroid hormone medication. A logistic regression analyses was performed with the significant variables from Table 3 as covariates. Treatment with calcium in combination with vitamin D3 and bisphosphonates were, however, not entered in the analyses since it is likely that the medication was given to patients with known fractures or risk factors of fracture. The dependent variable was a vertebral fracture (yes or no). After logistic regression only age remained significantly associated with vertebral fractures. Area under the ROC curve for age was 0.69, confidence interval (0.59 to 0.78).

**Table 3:** Comparisons between female SLE patients with and without radiological vertebral fractures.

	No vertebral compression (n= 107)	At least one vertebral compression (n = 43)
Patient age (years)	46 (20 to 79) ***	55 (28 to 82)
Postmenopausal n(%)	49 (46) # **	32 (76)
DI	2(0 to 8) *	3 (0 to 11)
Calcium and vitamin D n(%)	52 (49) # *	29 (67)
Bisphosphonates n (%)	10 (9) # *	11 (26)
BMD Total hip (g/cm^2^)	0.94 (0.14) *	0.88 (0.16)
BMD Femoral neck(g/cm^2^)	0.91 (0.14) *	0.86 (0.15)
Patients with peripheral fracture *n *(%)	10 (10) # **	12 (28)

Only significant variables are shown. BMD, bone mineral density, DI, Systemic Lupus International Collaborative Clinics/American Collage of Rheumatology Damage Index

*P ≤ 0.05, **P ≤ 0.01, ***P ≤ 0.001 vs. women with any compression fracture by t-test.

#χ^2^-test used to compare categorical variables.

### Risk factors of thoracic vertebral fractures

Thirteen patients had one or several vertebral fractures in L2 to L4, the area measured by DXA. Presence of vertebral compression fractures in this location may influence the DXA measurements. As a consequence new analyses were made where women with no vertebral fractures (*n *= 107) were compared to women with at least one vertebral fracture, but none in L2 to L4 (*n *= 30). Only patient age (*P *= 0.006) and DI (*P *= 0.04) differed significantly between the groups. High age remained associated with vertebral fractures in a logistic regression, area under ROC 0.64, confidence interval (0.53 to 0.76).

### Risk factors of lumbar vertebral fracture

Women with vertebral compression fractures in the lumbar spine (*n *= 15) were compared with women without fractures in the lumbar spine (*n *= 135). BMD in the total hip remained associated with lumbar fracture. Area under ROC was 0.68, confidence interval (0.52 to 0.84).

### Two or more vertebral fractures

Regardless of fracture localisation (lumbar, thoracic or both), patients with at least two vertebral fractures were compared with patients with one or no fracture in logistic regression analyses. Both BMD in total hip and age remained associated with prevalent fractures (two or more). The area under ROC was 0.8, with a confidence interval of 0.7 to 0.9.

### Vertebral fractures and normal BMD

Seventeen (40%) of the patients with vertebral deformities had normal BMD in all measured locations. There were three peripheral fractures reported from this group which had a median of one (1 to 10) compression, median age of 47 (28 to 59) years and disease duration of 12 (2 to 41) years. Having ever used corticosteroids was statistically equally common (*P *= 0.84) in women with vertebral fractures and normal BMD 14/17 (84%) compared to those with vertebral fracture and low BMD in at least one measured location 22/26 (82%).

## Discussion

Data from studies in healthy populations suggest that a majority of vertebral compressions are subclinical [8,21].

Twenty-nine percent of the patients in our study had at least one prevalent, radiological, vertebral compression. Eighty-eight percent of these fractures were asymptomatic. This can be compared with a slightly younger Dutch SLE population, mean age 41 years, where 20% of the patients had at least one prevalent vertebral fracture [22] whereas the prevalence was 21% in a premenopausal cohort of SLE women in Brazil [2]. A high prevalence, 20.4%, of asymptomatic vertebral fractures was also found in Chinese women with SLE [23]. The majority of compressions recorded in our study were mild and located in the thoracic spine confirming previous results in SLE studies [22] and in studies on post-menopausal women, with other inflammatory conditions, on long-term glucocorticosteroid therapy [24]. In the general population there are indications of compression fractures being more abundant in the thoracolumbar junction (Th11 to L2) [4,25]. Why fracture location seems to differ between different populations is not clear.

Without possibility of a direct comparison, a study on the Swedish general population showed a 7.2% 10-year probability of morphometric vertebral fracture in women 50 years of age increasing to 26.8% in women 80 years of age [8]. The percentage of SLE patients with prevalent vertebral fractures in our study increased per decade of life from 6% in the third decade to 54% in the eighth indicating that SLE patients are at high risk of developing compression fractures.

Forty percent of the patients with vertebral compressions in our study displayed normal BMD in all measured locations. Li *et al. *found that 30% of the SLE patients with asymptomatic vertebral fracture had a normal BMD [23]. Similar results have been found in SLE patients by Borba *et al. *[2] who found no BMD difference between premenopausal SLE patients with or without vertebral fractures. Lee *et al. *[13] found that 50% of patients with self reported fragility fractures had BMD z-score >-1 at hip and lumbar spine whereas Yee *et al*. [26] found normal BMD in few (9%) SLE patients with self reported fragility fractures. In a Swedish study on the general population, women with clinical vertebral fractures had significantly lower BMD in hip, lumbar spine and forearm compared with age-matched controls without a history of fracture [27]. It seems that clinical vertebral fractures more often are osteoporotic than prevalent fractures.

We found that high age was associated with fractures in Th4 to L4 while low BMD in total hip was a determinant of fracture in the lumbar spine. Age and BMD total hip together were strong determinants of repeated prevalent fractures. Mendoza-Pinto *et al*. [28] showed similar results in a Mexican SLE cohort, age and BMD total hip being associated to vertebral fractures. We believe that low total hip BMD may predict vertebral fractures better than lumbar spine BMD because there are several sources of error in the measurement of lumbar spine BMD. A false high BMD in lumbar spine can be caused by arterial calcifications, osteoarthritis or prevalent vertebral compression fractures in the measured area. Since SLE patients are at high risk of cardiovascular disease, arterial calcifications should be more pronounced in a SLE cohort at a given age compared to the general population.

Our results show that a large proportion of SLE patients get vertebral fractures despite normal BMD. Since some SLE patients are at high risk of thrombosis, impaired microcirculation in bone could damage bone cell viability and subsequently the possibility to repair trabecular damage. Mechanisms leading to impaired bone strength could also be initiated by autoantibodies directed against substances necessary for healthy bone remodelling. Frequent medication with glucocorticosteroids could be another explanation for fractures despite normal BMD. It has been shown that glucocorticosteroids induce trabecular thinning and affect osteocyte number and function. This could reduce vertebral compression strength more at a given BMD compared to the strength of vertebrae in aging or postmenopausal population [7,29]. Despite the known side effects of glucocorticosteroids we did not find any association between vertebral fracture and corticosteroids in our study. We have previously described a lack of association between glucocorticosteroids and low BMD in SLE patients [14]. One possible explanation is that systemic inflammation, also known to accelerate bone loss, is down regulated by corticosteroids. In a systemic inflammatory disease like SLE, glucocorticosteroids could be beneficial regarding bone loss in some individuals whereas it could decrease BMD and facilitate evolvement of fracture in higher doses or in more corticosteroid susceptible individuals.

When evaluating conventional radiographs for vertebral compression fractures it is possible to use morphometric or semiquantitative methods. The Genant method is a generally accepted semiquantitative method which is used in other SLE studies [22], studies on corticosteroid induced osteoporosis [24] as well as recent studies on medication against osteoporosis [30,31]. The advantage of Genant's method is that the radiologist adds accuracy to the evaluation by looking for qualitative features that are helpful in identifying fractures. Black *et al. *[32] have compared methods for defining prevalent vertebral deformities in osteoporosis. They conclude that patients with deformities rated as mild (grade 1) had clinical criteria and BMD midway between patients with no fractures and those with grade 2 or 3 fractures, indicating a continuum of vertebral pathology. We therefore believe the used, semiquantitative method is valid.

A limitation in our study is the absence of information about vitamin D status in our patient cohort, a variable of importance to bone mineralisation. Fifty-four percent of the SLE patients were taking supplements of calcium and vitamin D3. We therefore assume that they had no deficiency of vitamin D3. We also lack hereditary information of low energy fractures, information about height reduction and data on alcohol consumption. We do not know when the vertebral fractures have been acquired and risk factors may therefore have changed over time.

Besides BMD there are other factors influencing the risk of vertebral fractures such as bone dimensions, bone and intervertebral disc quality, micro-architecture of the bone, spine loading, and neuronal and muscle function [33]. Whether SLE inflammation affect these or other spine qualities in any particular way is not known.

## Conclusions

Vertebral compression fractures are common but seldom diagnosed in patients with SLE regardless of treatment with glucocorticosteroids or not. High age and low BMD in the total hip are the most important risk factors associated with fracture. There may be disease-specific factors interacting and affecting bone strength in SLE. Therefore, in clinical guidance of which patients should be sent to vertebral x-ray or receive anti-osteoporotic therapy, SLE itself should be considered to add risk to already known general risk factors of osteoporosis and fracture.

## Abbreviations

ACR: American College of Rheumatology; BMD: bone mineral density; BMI: body mass index; CRP: C-reactive protein; DXA: dual energy x-ray absorptiometry; DI: Systemic Lupus International Collaborative Clinics/American College of Rheumatology Damage Index; ESR: erythrocyte sedimentation rate; GFR: glomerular filtration rate; HRT: hormone replacement therapy; ROC: receiver operating characteristic; SD: standard deviations; SERM: selective estrogen receptor modulator; SLE: systemic lupus erythematosus; SLEDAI-2K: SLE disease activity index-2K; WHO: World Health Organization

## Competing interests

The authors declare that they have no competing interests.

## Authors' contributions

KA conceived the study, participated in its design and coordination, performed most of the statistical analyses and drafted the manuscript. SH evaluated all radiographs and contributed with knowledge about vertebral pathology. CO participated in coordination of DXA measurements and interpretation of data. HC participated in study design, interpretation of data and revision of the manuscript. HF-d'E participated in study design and coordination, the interpretation of statistical analyses and revision of the manuscript. All authors read and approved the final manuscript.
